# Supplementary material for: Reptile-like physiology in Early Jurassic stem-mammals
Source: Nat Commun. 2020 Oct 12;11:5121. doi: 10.1038/s41467-020-18898-4 (PMC7550344; doi:10.1038/s41467-020-18898-4)
Supplement: Supplementary file 1 — Supplementary Information [file 41467_2020_18898_MOESM1_ESM.pdf]

Supplementary Information

Reptile-like physiology in Early Jurassic stem-mammals

Newham et al.

Contents:

Supplementary Tables……………………………………….………………………………........3

Supplementary Table 1. Results of quantitative analyses of precision between three independent observers for the cementum increment counts of fossil mammals studied here……………...…….3

Supplementary Table 2. Results of measuring femur length and nutrient foramen radius in μCT data for 11 extant mammal taxa and *Morganucodon*, with resulting estimates of blood flow index Q_i…...4

Supplementary Figures……………………………………...……………………….……………5

Supplementary Figure 1. Example of splitting and coalescence of cementum increments in specimen NHMUK PV M 104138……………………………...…………………………..…5

Supplementary Figure 2. Common biological and physical features, and diagenetic fabrics, encountered in tomographic data of fossil cementum……………………………………..……..7

Supplementary Figure 3. Comparison between distributions of cementum increment counts estimated by three independent observers…………………………..…………………………..…..……9

Supplementary Figure 4. Differences between captive and wild maximum lifespans for extant taxa for which both values are known ……………………………………..……………..…...…10

Supplementary Figure 5. Lifespan and metabolic estimates of *Morganucodon* and *Kuehneotherium* based on maximum captive lifespan of extant mammals and reptiles…….……………………11

Supplementary Figure 6. The relationship between maximum captive lifespan and msSMR, and maximum captive lifespan and growth rate constant *K*, in mammals and reptiles...........................13

Supplementary Notes…………………………………………………………………………………..…15

Supplementary Note 1: Taxonomic position of *Morganucodon* and *Kuehneotherium*……………………………………………..……………………….…...….15

Supplementary Note 2: Further information on Glamorgan fissure fill and specimen choice……………………………………………………………………………...…………15

Supplementary Note 3: Eruption sequence and timing……………..……………………………..17

Supplementary Note 4: Comparisons between fossil lifespan estimates and captive lifespans of extant taxa…………………………………………………………………………………………19

Supplementary References………………...…………………………………………………………22

Supplementary Tables

Supplementary Table 1 | Results of quantitative analyses of precision between three independent observers for the cementum increment counts of fossil mammals studied here, *Morganucodon* and *Kuehneotherium*, and those of ten previous studies of extant mammals with comparable age ranges. *CV* – coefficient of variation.

**Study**	**Taxon**	***n***	**Maximum age (years)**	**mean CV**
Newham et al. (this study)	*Morganucodon*	34	14	9.32
Newham et al. (this study)	*Kuehneotherium*	27	9	4.89
Grau et al.[1]	*Procyon lotor*	54	9	20.6
Gasawey et al.[2]	*Alces alces*	72	9	14.2
Klevezal and Pucek[3]	*Bison bonasus*	45	21	20.57
Kay and Cant[4]	*Macaca mulatta*	65	24	29.34
Cederlund et al.[5]	*Capreolus capreolus*	74	9	30.5
Bodkin et al.[6]	*Enhydra lutris*	14	14	26.24
London et al.[7]	*Canis lupus*	12	6.8	25.92
Christensen-Dalsgaard et al.[8]	*Ursus maritimus*	32	15	15.2
Pasda[9]	*Rangifer tarandus*	63	16	19.4
Perez-Barberia et al.[10]	*Cervus elaphus*	164	17	16.22

Supplementary Table 2 | Results of measuring femur length and nutrient foramen radius in µCT data for 11 extant mammal taxa and *Morganucodon*, with resulting estimates of blood flow index Q_i.

Taxon	Average femur length (cm)	Average foramen radius (cm)	Average Q_i (mm^3)
Apodemus flavicolis	2.15	0.082	3.15E-05
Micromys minutus	1.1	0.062	1.47E-05
Microtus levis	1.43	0.071	2.26E-05
Mus musculatus	1.32	0.073	2.38E-05
Peromyscus truei	1.75	0.063	1.10E-05
Sorex minutissimus	0.48	0.045	1.00E-05
Sicista betulina	1.04	0.071	2.59E-05
Sorex araneus	0.82	0.044	4.89E-06
Myodes rutilus	1.17	0.066	1.61E-05
Neomys fodiens	1.04	0.068	2.28E-05
Sorex minutus	0.56	0.042	6.37E-06
Morganucodon	1.25	0.026	3.83E-07

Supplementary Figures

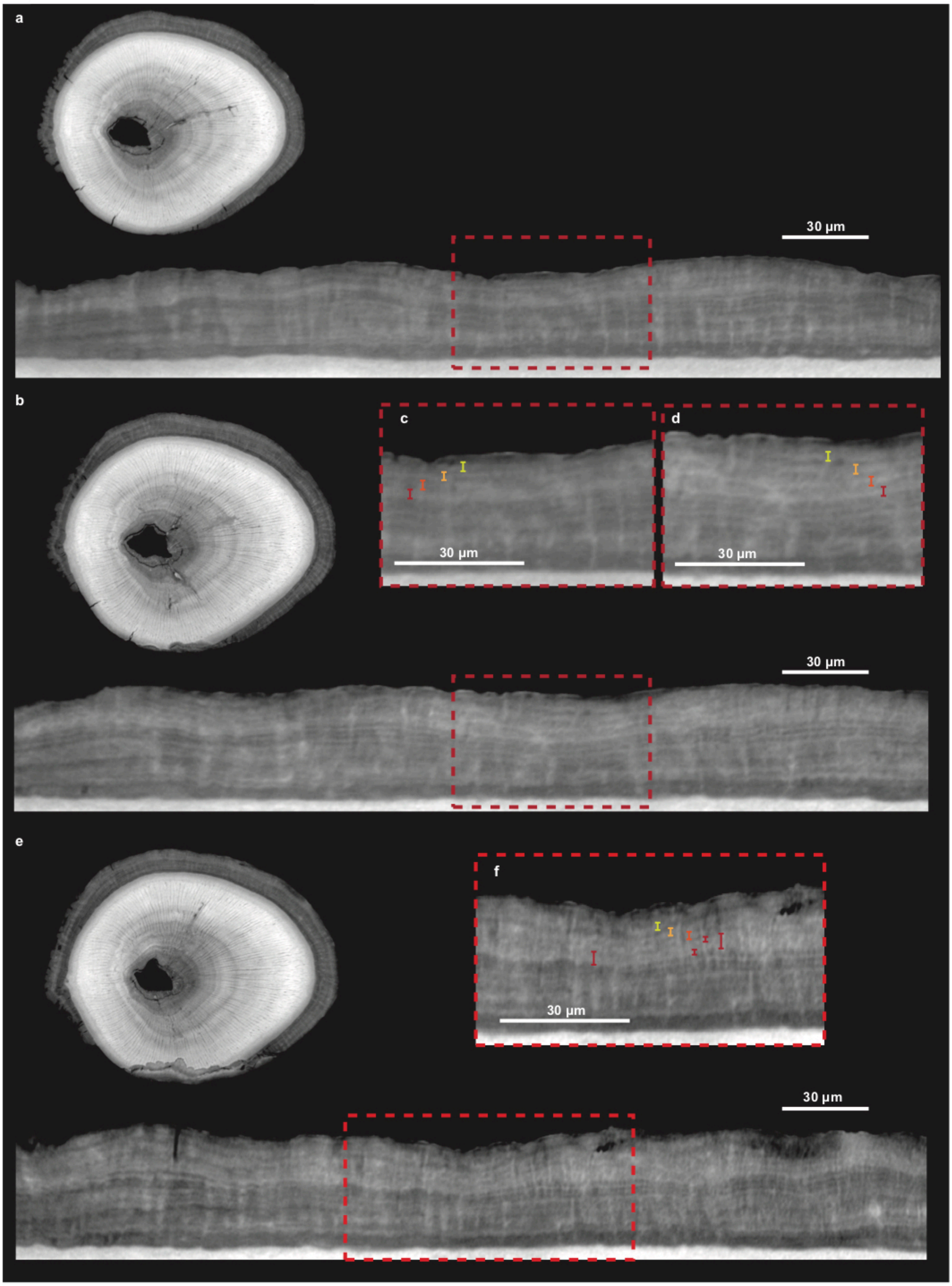

Supplementary Figure 1 | Example of spliting and coalescence of cementum increments in specimen NHMUK PV M 104138. a, Transverse PPC-SRµCT slice selected from region

of the cementum closest to the crown. Inset to the left is the entire slice, and the straightened portion represents the region of highest increment contrast from this slice. **b,** Transverse PPC-SRµCT slice 100 µm towards the root apex relative to **a**. **c,** Detail from **a** highlighted by dashed red box, with four outermost higher density (lighter coloured) cementum increments annotated with coloured bracketed lines. **d**, Detail from **b** highlighted by dashed red box. The same increments imaged in **a** and **c** are annotated with the same coloured bracketed lines. However, they are more clearly defined in **b** and **d**, with the innermost two annotated increments **(**dark red and orange bracketed lines) coalescing in **a** and **c**. **e,** Transverse PPC-SRµCT slice 100 µm towards the root apex relative to **b. f**, Detail from **e** highlighted by dashed red box. While the four outermost increments shown in the other PPC-SRµCT slices are also represented here, the innermost increment (dark red bracketed lines) has split, creating two accessory increments. All straightening performed using the "straighten" tool in ImageJ/Fiji[11].

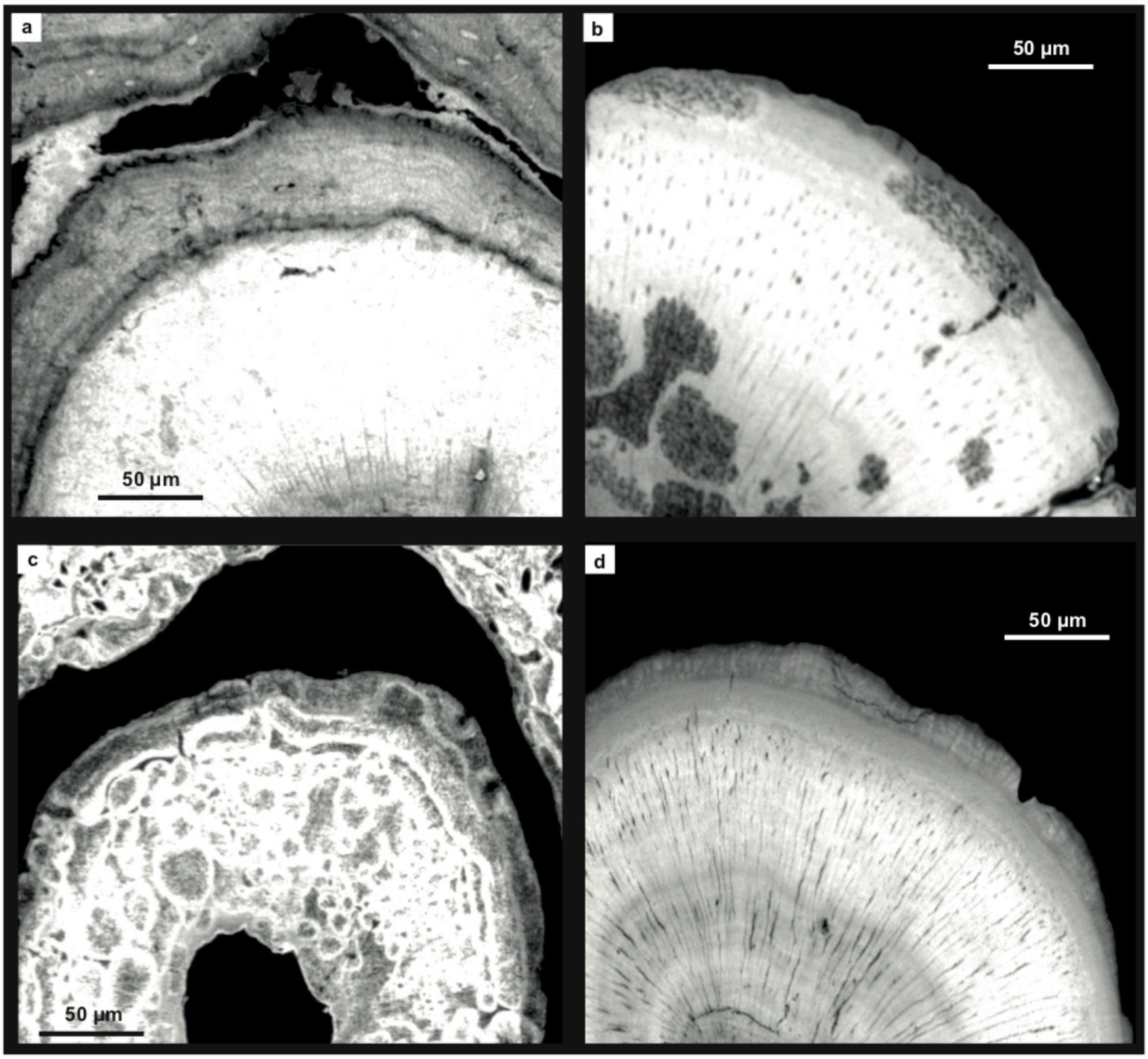

Supplementary Figure 2 | Common biological and physical features, and diagenetic fabrics, encountered in tomographic data of fossil cementum. a, Substantial variation in the thickness of individual cementum increments in the anterior root of the m2 specimen NHMUK PV M 104129. **b,** Discrete dark, less dense regions of diagenetic alteration within the root of NHMUK PV M 96086, a specimen of otherwise excellent dentine and cementum preservation. **c,** Globular diagenetic fabrics have adulterated virtually all microstructure in the anterior root of the m1 specimen NHMUK PV M 95809, though it may still be possible to separate dentine and cementum. **d**, Physical damage to the cementum tissue has removed outer increments in discrete regions of the cementum of the anterior root of NHMUK PV M

96273. The dentine has been over-saturated (white) by decreasing the dynamic range in imageJ/Fiji[11] in order to improve the visibility of the cementum.

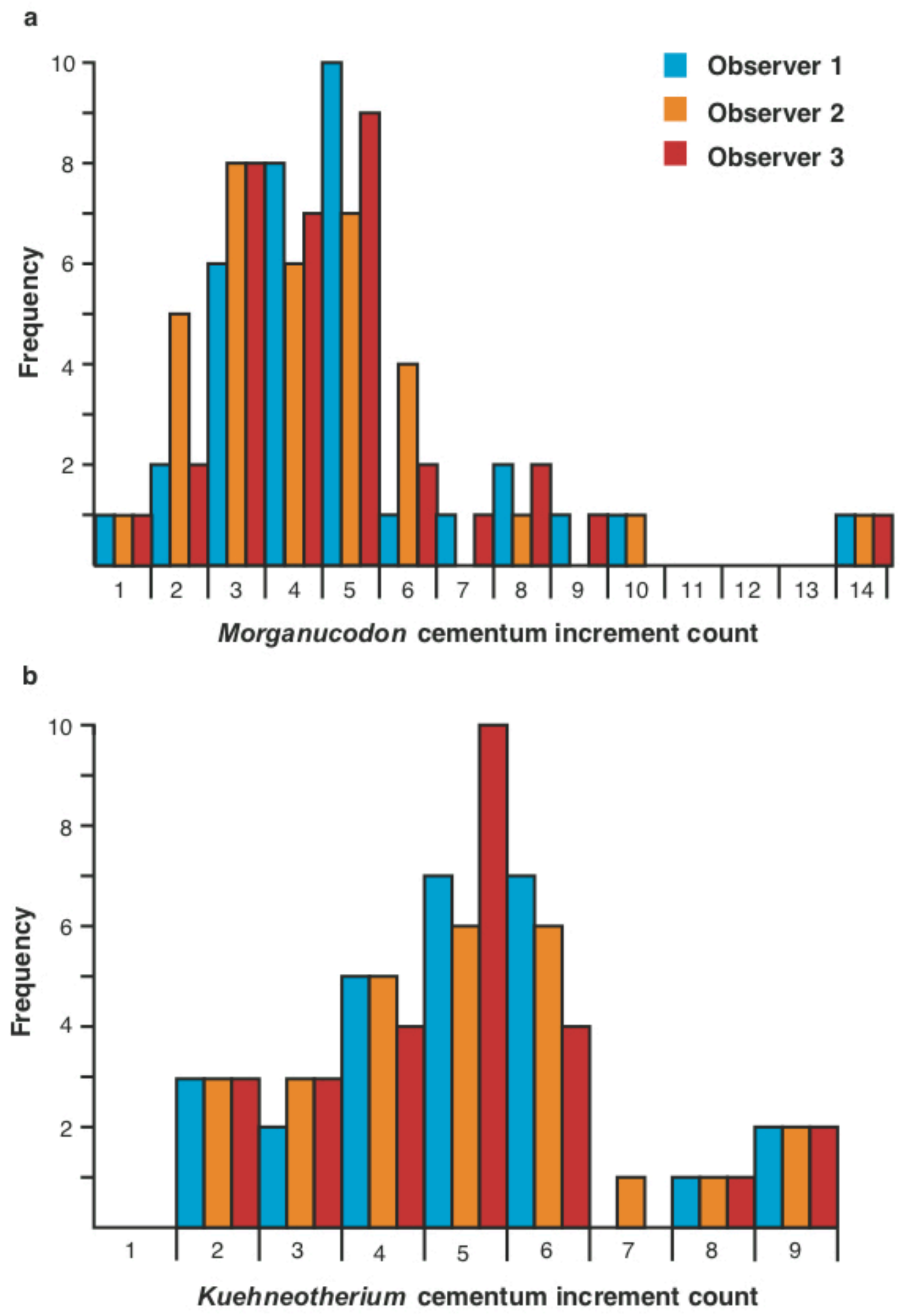

Supplementary Figure 3 | Comparison between distributions of cementum increment counts estimated by three independent observers. a, Distributions of increment counts estimated for *Morganucodon.* **b,** Distributions of increment counts estimated for *Kuehneotherium.* Source data are provided as a Source Data file.

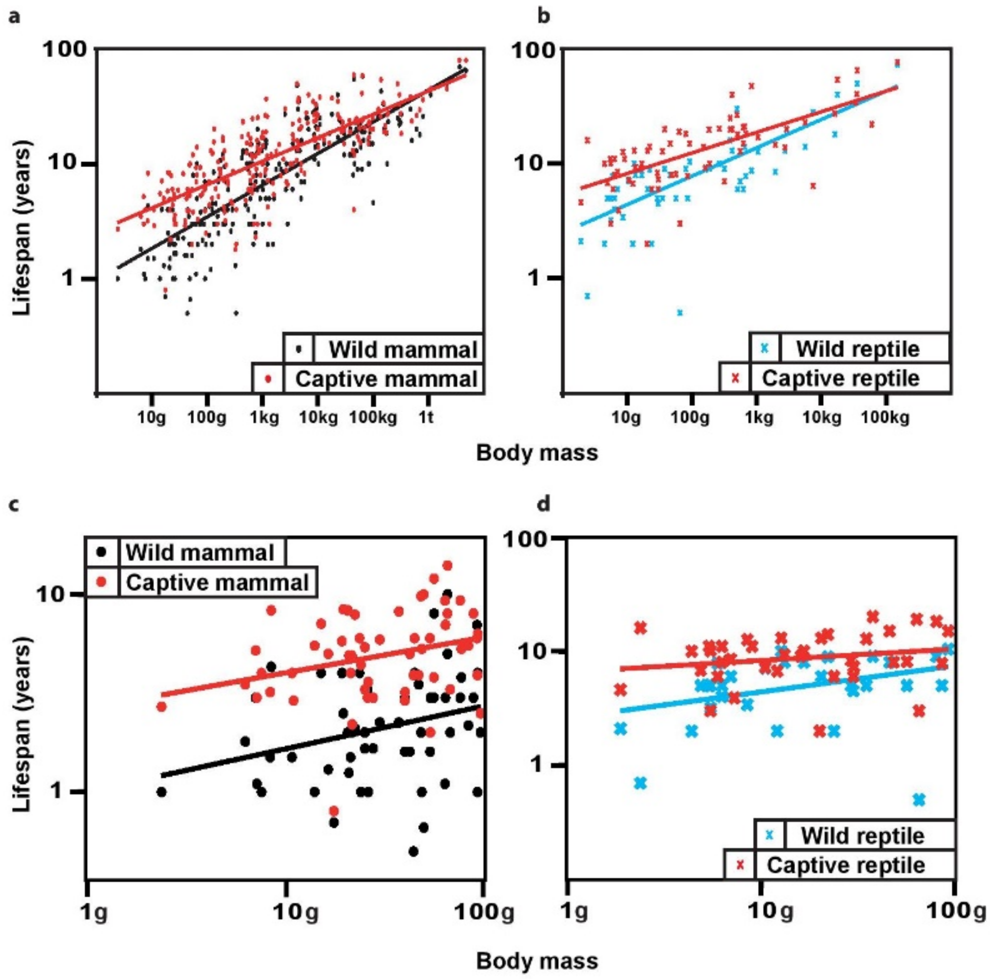

Supplementary Figure 4 | Differences between captive and wild maximum lifespans for extant taxa for which both values are known. **a**, Log_{10} biplot of mean body mass (g) against maximum wild vs maximum captive lifespan for 244 extant mammals. **b**, Log_{10} biplot of mean body mass (g) against maximum wild vs maximum captive lifespan for 68 extant reptiles. **c**, Log_{10} biplot of mean body mass (g) against maximum wild vs maximum captive lifespan for 56 extant mammals with body masses below 100 g. **d**, Log_{10} biplot of mean body mass (g) against maximum wild vs maximum captive lifespan for 38 extant mammals with body masses below 100 g. Source data are provided as a Source Data file.

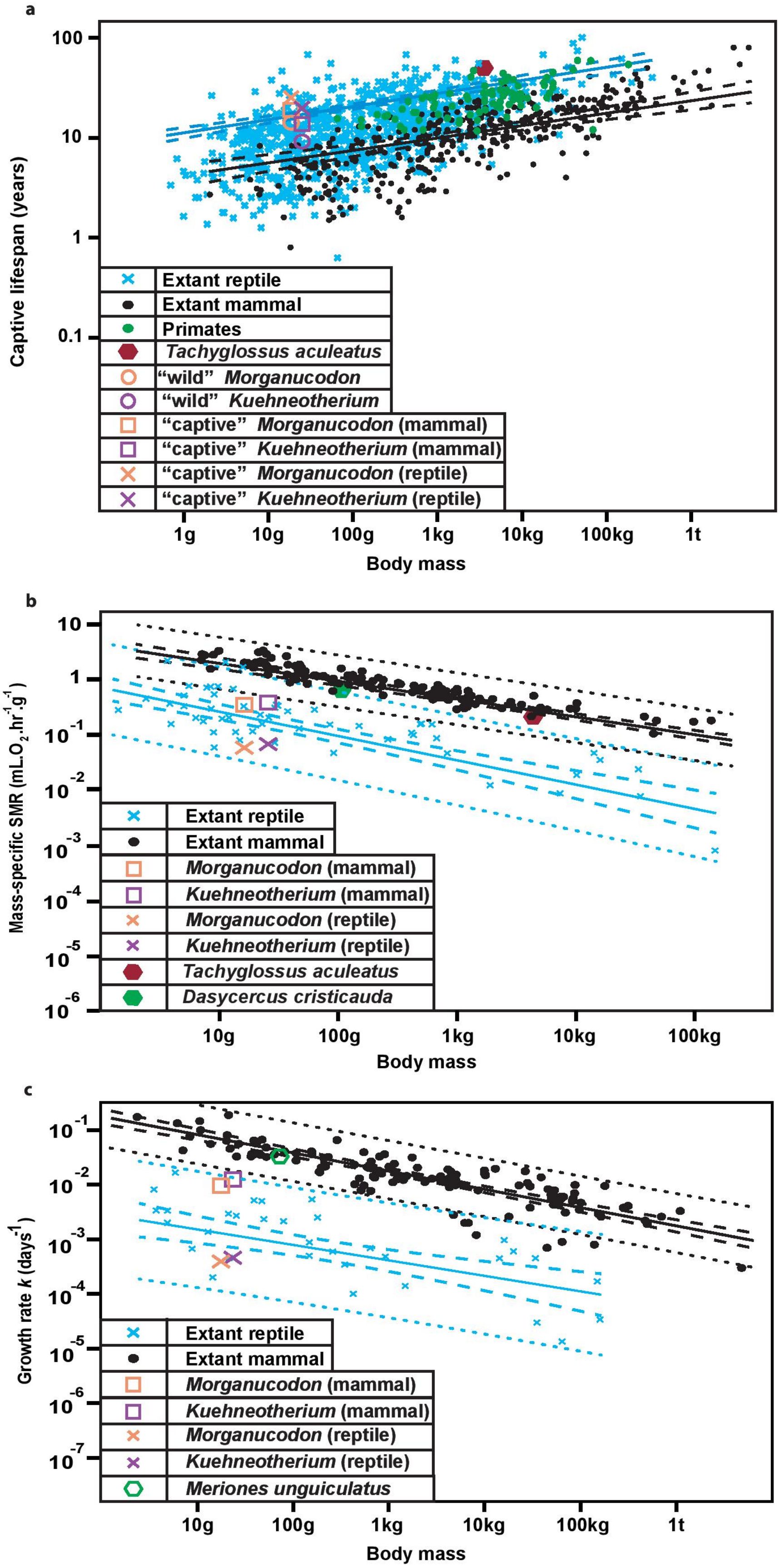
a
Captive lifespan (years)
100
10
1
0.1
Extant reptile
Extant mammal
Primates
Tachyglossus aculeatus
"wild" Morganucodon
"wild" Kuehneotherium
"captive" Morganucodon (mammal)
"captive" Kuehneotherium (mammal)
"captive" Morganucodon (reptile)
"captive" Kuehneotherium (reptile)
1g
10g
100g
1kg
10kg
100kg
1t
Body mass
b
Mass-specific SMR (mL.O2.hr-1.g-1)
10
1
10-1
10-2
10-3
10-4
10-5
10-6
Extant reptile
Extant mammal
Morganucodon (mammal)
Kuehneotherium (mammal)
Morganucodon (reptile)
Kuehneotherium (reptile)
Tachyglossus aculeatus
Dasycercus cristicauda
10g
100g
1kg
10kg
100kg
Body mass
c
Growth rate k (days-1)
10-1
10-2
10-3
10-4
10-5
10-6
10-7
Extant reptile
Extant mammal
Morganucodon (mammal)
Kuehneotherium (mammal)
Morganucodon (reptile)
Kuehneotherium (reptile)
Meriones unguiculatus
10g
100g
1kg
10kg
100kg
1t
Body mass

Supplementary Figure 5 | Lifespan and metabolic estimates of *Morganucodon* and *Kuehneotherium* based on maximum captive lifespan of extant mammals and reptiles. a, Log_{10} biplot of mean body mass (g) against maximum captive lifespan (years) for extant mammals (n = 458), extant non-avian reptiles (n = 801), and fossil mammaliaforms. **b,** Log_{10} biplot of mean body mass (g) against mass specific standard metabolic rate (msSMR; $mL.O_2.hr^{-1}.g^{-1}$) for extant mammals (n = 117) and extant reptiles (n = 55), and estimates for fossil mammaliaforms. **c,** Log_{10} biplot of mean body mass (g) against post-natal growth rate constant K ($days^{-1}$) for extant mammals (n = 115) and extant reptiles (n = 33) and estimates for fossil mammaliaforms. PGLS regression lines are shown for extant mammals (black) and extant reptiles (blue), 95% confidence intervals are represented by dashed lines, 95% predictor intervals by dotted lines. Source data are provided as a Source Data file.

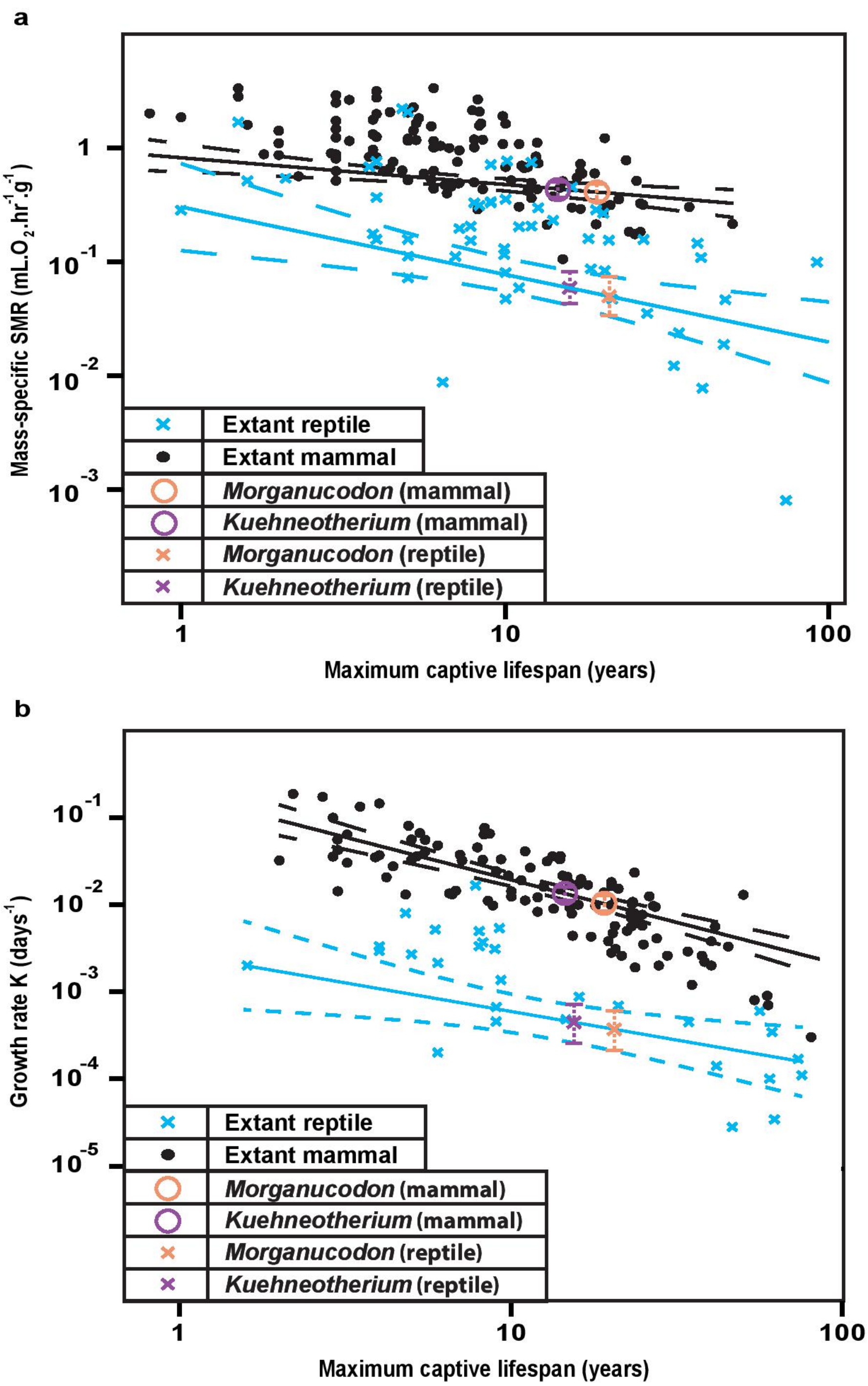

Supplementary Figure 6 | The relationship between maximum captive lifespan and msSMR, and maximum captive lifespan and growth rate constant *K*, in mammals and reptiles. a, Log_{10} biplot between maximum captive lifesan (years) and mass-specific standard metabolic rate (msSMR; $mL.O_2.hr^{-1}.kg^{1}$) for extant mammals (n = 117) and reptiles (n = 55). **b,** Log_{10} biplot between maximum captive lifespan (years) and post-natal growth rate

constant *K* ($days^{-1}$) for extant mammals (n = 115) and reptiles (n = 29). PGLS regression means for each clade (black lines for mammals, blue lines for reptiles, dashed lines denote 95% confidence intervals) are used to estimate msSMR and *K* for mammaliaforms *Morganucodon* and *Kuehneotherium*, with dashed brackets denoting their 95% confidence intervals. Source data are provided as a Source Data file.

Supplementary Notes

Supplementary Note 1: Taxonomic description of *Morganucodon* and *Kuehneotherium*

Order MORGANUCODONTA Kermack, Mussett and Rigney, 1973

Family MORGANUCODONTIDAE Kühne 1958

Genus and species MORGANUCODON WATSONI Kühne, 1949

Family KUEHNEOTHERIIDAE Kermack, Kermack and Mussett, 1968

Genus and species KUEHNEOTHERIUM PRAECURSORIS Kermack, Kermack and Mussett, 1968

The phylogenetic relationship of *Morganucodon* and *Kuehneotherium* is shown in Fig. 2. The position of *Morganucodon* at the base of Mammaliaformes (Node 4) has remained stable between multiple studies[12,13,14,15,16]. We have also chosen to position *Kuehneotherium* at the Mammaliaformes node in Fig. 2, as its lack of diagnostic post-cranial material makes its phylogenetic placement uncertain and poorly supported amongst studies[15,16,17] However, it is generally placed either within a clade including *Morganucodon*[16,17] or further crownward than *Morganucodon* based mainly based on the triangulation of its molars[15,16,17]

Supplementary Note 2: Further information on Glamorgan fissure fill and specimen choice.

The *Morganucodon* and *Kuehneotherium* specimens used for this study are all from the Hettangian-Sinemurian age fissure fills of Glamorgan[12,18]. The relevant fissures are from two quarries, Pant and Pontalun (now Lithalun), and the material was collected in the 1950s to 1970s. All the *Morganucodon* specimens, and a few *Kuehneotherium* specimens, are from

Pontalun 3 fissure, but most of the *Kuehneotherium* specimens are from Pant quarry, fissures 2 and 4, where it is more abundant. The origin fissure and more information on the specimen elements are summarised in Supplementary Data File 2.

The fissure material is disarticulated, and individual elements are usually broken or cracked during deposition and preservation. However, the individual preservation is generally good in Pontalun 3, and the material has been prepared without acid. The first stage of the project, therefore, focused on *Morganucodon watsoni* from this fissure, particularly the lower second molars, as they have large roots and are easily identified and scanned. The project was then expanded to include other *Morganucodon* isolated teeth and dentulous specimens from Pontalun 3. *Morganucodon* dentary specimens are more likely to retain teeth than those of *Kuehneotherium*, due to the expanded root apices in the *Morganucodon* ultimate premolars and the molars. The *Morganucodon* dentulous specimens proved to have the best preservation of the cementum in general, especially when the crowns were intact at the time of fossilisation, possibly due to the cementum and root canal not being exposed to taphonomic processes. Although all the *Morganucodon* specimens are from the same fissure, they cannot be regarded as in any way a cohort population, as the specimens occurred scattered throughout the large fissure in small pockets, and were probably washed in from the surrounding area during rain storms following forest fires[17,18].

Kuehneotherium specimens are rare in Pontalun 3 fissure, and so it was necessary to mainly choose specimens from the two Pant fissures where they are more abundant. The better-preserved specimens were selected, whether uppers or lowers. Dentulous specimens are rare in *Kuehneotherium*, and most of the cementum counts are from isolated teeth. Only those teeth which could be identified as *Kuehneotherium* could be used, which limited the choice to the molars and the distinctive ultimate and penultimate lower premolars, which have a double distal accessory cusp[19,20]. As for *Morganucodon*, the *Kuehneotherium*

specimens are not a cohort population, and they are from three different fissures. Also, Pant 4 has a different faunal composition to that of Pant 2 and Pontalun 3, so, although close geographically to Pant 2, it is obviously from a different depositional event. Pontalun 3 and Pant 2 have a limited tetrapod fauna comprising *Morganucodon, Kuehneotherium,* and *Gephyrosaurus*, whereas Pant 4 has a much wider fauna including *Clevosaurus, Oligokyphus* and haramiyids[18].

Supplementary Note 3: Eruption sequence and timing

As noted in the main text, establishing individual ages from several teeth along *Morganucodon* lower jaws provides information on the tooth eruption sequence and timing. Eight scanned dentaries provided information (Table 1). There are several specimens with tooth row increment counts covering p1 to m2: For example, NHMUK PV M 95790 for p1 to p3; NHMUK PV M96413 for p3 to m1; NHMUK PV M 96396 for p4 to m2 (Table 1). NHMUK PV M 96413 and NHMUK PV M 96396 also show readable lines of arrested growth (LAGs) in the dentary bone, which provide the same count as their p3 to m1 and p4-m2 teeth respectively. This indicates that p1 to m2 erupted within one year of each other in *Morganucodon*.

There is also a specimen (NHMUK PV M 27312) with an increment count of one (Supplementary Data File 1). This specimen has the deciduous fourth premolar in situ, with signs of the developing permanent third and fourth premolars forming, but also the first molar *in situ* and m2 in the process of erupting. This shows that m1 erupted before p3 and 4, and all within one year of life. In the mammaliaform *Docodon victor* the third premolar p3 is the earliest tooth position for premolar replacement[21], but that level of differentiation cannot yet be determined in *Morganucodon*.

Three specimens (NHMUK PV M 96396; NHMUK PV M 104129; NHMUK PV M 104130) show that the third molar erupted during the year after the permanent p1-m2 erupted. One specimen (NHMUK PV M 95790) has cementum increment counts indicating that the permanent final incisor i4, and the canine, erupted during the year after the eruption of the first three permanent premolars. We therefore conclude that the ultimate incisor, canine and the third molar erupted during the year following the eruption of p1 to m2 in *Morganucodon*. We do not have information on eruption timing of more anterior incisors, or the fourth molar. The fifth molar is only very occasionally present.

The eruption of the final incisor and canine after the premolars may be a plesiomorphic feature in *Morganucodon* and does not follow the general antero - posterior pattern seen in later therians. In the Late Jurassic dryolestids[22], tooth replacement takes place in two waves; with i2, i4, p1, and p3 in the first series and i1, i3, c, p2, and p4 in the second. However, this is also different from the pattern seen here in *Morganucodon*, where the ultimate incisor and canine come in after the permanent premolars.

Unfortunately, there are no available tooth rows of *Kuehneotherium* with cementum increment counts. Dentulous specimens are rare and the three specimens in the UMZC collection are not suitable for scanning for cementum annuli, as they were previously mounted in plaster and dissected to reveal the tooth roots. *Kuehneotherium* has a longer tooth row than *Morganucodon* with six premolars and six molars[23]. A difference from *Morganucodon* is that there is evidence from specimens of juvenile *Kuehneotherium* dentaries[23] that the canine, ultimate premolar, p6, and third molar all erupted at about the same time, which suggests that the canine erupted relatively earlier in *Kuehneotherium* than in *Morganucodon*.

The loss of the anterior permanent postcanines has been noted in *Morganucodon* jaws[24], and is assumed to proceed along the tooth row with age. In some dentaries, the first

premolar appears to be lost relatively early and is only represented by a small, indistinct, resorbed root just posterior to the canine. Accordingly, it is not certain if the timing, and degree, of loss of the anterior premolars is variable in individuals, but this question is part of a current study. Specifically, the specimen NHMUK PV M 95790 with cementum annuli counts for i4 to p3, obviously has not shed the anterior premolars, despite a minimum age of eight years.

Supplementary Note 4: Comparisons between fossil lifespan estimates and captive lifespans of extant taxa

In our primary study, we compare the lifespan estimates for our fossil taxa with maximum known wild lifespans for extant mammals and reptiles. Environmental and ecological pressures upon wild populations mean that wild individuals rarely live until the maximum possible age for a taxon, and this is shown when we compare the maximum known wild lifespan and the maximum known captive lifespan for individual mammal (n = 244) and reptile (n = 68) taxa (Supplementary Fig. 4). ANCOVA comparisons show that the wild and captive regression slopes are significantly different for both mammals ($p<0.001$) and reptiles ($p = 0.048$). Similar to previous studies[25,26], we find that small bodied taxa, both mammals and reptiles, show larger relative per-taxon lifespan increases in captivity compared to larger taxa (Supplementary Fig. 4). ANCOVA comparison of extant taxa under 100g shows that the wild and captive regression slopes are significantly similar for both mammals ($p = 0.77$) and reptiles ($p = 0.37$) and that captive maximum lifespan exceeds wild maximum lifespan for the same taxon by an average of 3.43 years for mammals ($F = 67.14$; $p<0.001$), and 4.38 years for reptiles ($F = 15.61$; $p<0.001$). For small taxa, maximum captive lifespan may thus be more directly linked to the metabolic physiology of a taxon, compared to maximum wild lifespan, as it is less effected by extrinsic environmental/ecological factors that limit the

lifespan of individuals. However, our fossil samples cannot directly provide an estimate of captive lifespan.

In order to compare our fossil samples with the captive maximum lifespan estimates of extant mammals and reptiles, we have here used the average difference between per-taxon captive and wild lifespan for mammals and reptiles (respectively) to estimate a 'captive' potential maximum lifespan for our fossil taxa. This provides a maximum potential captive lifespan of either 17.4 (from the mammal difference) or 18.4 years (from the reptile difference) for *Morganucodon*, and 12.4 or 13.4 years respectively for *Kuehneotherium*.

When we compare these values to the maximum captive lifespans of extant mammals (n = 458) and reptiles (n = 801) (Supplementary Data File 3) using PGLS regression, estimates for both our fossil taxa are further above the mammalian regression line than any mammal below 1 kg (Supplementary Fig. 5a). Maximum captive lifespan estimates for *Morganucodon* are further above the mammalian regression line than any mammal apart from *Tachyglossus aculeatus* (Supplementary Fig. 5a) and a small number of primates, all larger than approximately 4kg. Phylogenetic ANCOVA shows the PGLS regression line for reptiles is statistically significantly higher than that for mammals ($F = 7.279$; $p = 0.0071$), as is the case for the wild lifespan dataset, showing reptiles have a longer captive lifespan at a given body mass than mammals.

Using PGLS regression between $\log_{10}$ transformed values of maximum transformed captive lifespan and standardised metabolic rate (msSMR) for extant mammals (n = 117; $\log_{10}$ msSMR = 0.237($\log_{10}$ captive lifespan) - 0.083; $r^2 = 0.41$; $p<0.001$) (Supplementary Fig. 6a), we estimate an msSMR value of 0.416 $mL.O_2.hr^{-1}.g^{-1}$ for *Morganucodon* and 0.45 $mL.O_2.hr^{-1}.g^{-1}$ for *Kuehneotherium.* From the reptile regression (n = 55; $\log_{10}$ msSMR = 0.597($\log_{10}$ captive lifespan) – 0.519; $r^2 = 0.36$; $p<0.001$), we estimate an msSMR value of 0.052 $mL.O_2.hr^{-1}.g^{-1}$ for *Morganucodon* and 0.063 $mL.O_2.hr^{-1}.g^{-1}$ for *Kuehneotherium*

(Supplementary Fig. 5b).When this data is PGLS regressed against body mass, the fossil mammaliaforms plot outside the living mammal confidence and prediction intervals, within the living reptile prediction intervals, and are distributed either side of the reptilian regression line, close to the confidence interval lines (Supplementary Fig. 5b).

Using PGLS regression between $\log_{10}$ transformed values of maximum transformed captive lifespan and post-natal growth rate (K) for extant mammals (n = 115; $\log_{10} K$ = -0.817($\log_{10}$ captive lifespan) – 0.908; r^2 = 0.67; p<0.001) (Supplementary Fig. 6b), we estimate a K of 1.164e^{-2} days^{-1} for *Morganucodon* and 1.512e^{-2} days^{-1} for *Kuehneotherium.* For reptiles (n = 29; $\log_{10} K$ = -0.656($\log_{10}$ captive lifespan) – 2.563; r^2 = 0.42; p<0.001) we estimate a K of 3.84e^{-4} days^{-1} for *Morganucodon* and 4.66e^{-4} days^{-1} for *Kuehneotherium* (Supplementary Fig. 5c).

When this data is PGLS regressed against body mass, all estimates for the fossil mammaliaforms fall outside of prediction intervals for the the mammal data, the majority fall within the reptile prediction intervals, and all are further below the mammal regression line than any extant mammal below approximately 8kg (Supplementary Fig. 5c).

In summary, whether using their raw/wild or adjusted 'captive' lifespan estimates, our fossil mammaliaforms are consistently predicted to have msSMR and K values lower than comparable extant endothermic mammals but within the range of values of extant ectothermic reptiles.

Supplementary References

1. Grau, G. A., Sanderson, G. C. & Rogers, J. P.1970. Age determination of raccoons. *The J. Wildlife Management*. **34**, 364-372 (1970).
2. Gasawey, W. C., Harkness, D. B. & Rausch, R. A. Accuracy of moose age determinations form incisor cementum layers. *J. Wildlife Management.* **42**, 558-563 (1978).
3. Klevezal, G. A. & Pucek, Z. Growth layers in tooth cementum and dentine of European bison and its hybrids with domestic cattle. *Acta Theriol.* **32**, 115-128 (1987).
4. Kay, R. F. & Cant, J. G. Age assessment using cementum annulus counts and tooth wear in a free-ranging population of *Macaca mulatta*. *Am. J. Primatology.* **15**, 1-15 (1988).
5. Cederlund, G., Kjellander, P. and Stålfelt, F. Age determination of roe deer by tooth wear and cementum layers-tests with known age material. In Csányi, S. & Ernhaft, J. (Eds). *Transactions of the 20th Congress of the International Union of Game Biologists, Gödöllö* (540-545). University of Agricultural Sciences. Gödöllö, Hungary (1991).
6. Bodkin, J.L., Ames, J.A., Jameson, R.J., Johnson, A.M. and Matson, G.M. Estimating age of sea otters with cementum layers in the first premolar. *J. wildlife management*. **61**, 967-973 (1997).
7. Landon, D.B., Waite, C.A., Peterson, R.O. and Mech, L.D. Evaluation of age determination techniques for gray wolves. *J. wildlife management.* **62**, 674-682 (1998).
8. Christensen-Dalsgaard, S.N., Aars, J., Andersen, M., Lockyer, C. and Yoccoz, N.G. Accuracy and precision in estimation of age of Norwegian Arctic polar bears (*Ursus maritimus*) using dental cementum layers from known-age individuals. *Polar Biology*. **33**, 589-597 (2010).
9. Pasda, K. Assessment of age and season of death of West Greenland reindeer by counting cementum increments in molars. *Documenta Archaeobiologiae.* **4**, 125-140 (2006).

10. Pérez-Barbería, F.J., Duff, E.I., Brewer, M.J. and Guinness, F.E. Evaluation of methods to age Scottish red deer: the balance between accuracy and practicality. *J. Zool*. **294**, 180-189 (2014).

11. Schneider, C. A., Rasband, W. S. & Eliceiri, K. W. NIH Image to ImageJ: 25 years of image analysis. *Nature Methods.* **9**, 671–675 (2001).

12. Gill, P. G., Purnell, M. A., Crumpton, N., Brown, K. R., Gostling, N. J. et al. Dietary specializations and diversity in feeding ecology of the earliest stem mammals. *Nature* **512**, 303–305 (2014).

13. Luo, Z. X. Transformation and diversification in early mammal evolution. *Nature* **450**, 1011–1019 (2007).

14. Rowe, T. B. Definition, diagnosis, and origin of Mammalia. *J. Vert. Paleontol.* **8**, 241–264 (1988).

15. Kielan–Jaworowska, Z., Cifelli, R. L., & Luo, Z. X. *Mammals from the age of dinosaurs: origins, evolution, and structure* (Columbia University Press, New York, United States, 2004).

16. Luo, Z-X, Kielan-Jaworowska, Z. & Cifelli, R. L. In quest for a phylogeny of Mesozoic mammals. *Acta Palaeontologica Polonica.* **47**, 1-78 (2002).

17. Kermack, K. A., Mussett, F., & Rigney, H. W. The lower jaw of *Morganucodon*. *Zool. J. Linn. Soc.* **53**, 87–175 (1973).

18. Whiteside, D. I., Duffin, C. J., Gill, P. G., Marshall, J. E., & Benton, M. J. The Late Triassic and Early Jurassic fissure faunas from Bristol and South Wales: stratigraphy and setting. *Palaeontol. Pol*. **67**, 257–287 (2016).

19. Kermack, D. M., Kermack, K. A., & Mussett, F. The Welsh pantothere *Kuehneotherium praecursoris. Zool. J. Linn. Soc.* **47**, 407–423 (1968).

20. Gill, P. G. Kuehneotherium *from the Mesozoic Fissure Fillings of South Wales.* Ph.D. thesis, Univ. Bristol (2004).

21. Schultz, J.A., Bhullar, B.A.S. & Luo, Z-X. Re-examination of the Jurassic Mammaliaform *Docodon victor* by Computed Tomography and Occlusal Functional Analysis. *Journal of Mammalian Evolution*. **26,** 9-38. (2019).

22. Martin, T. Tooth replacement in Late Jurassic Dryolestidae (Eupantotheria, Mammalia). *Journal of Mammalian Evoutionl.* **4**, 1–18. (1997).

23. Gill, P. G. Kuehneotherium *from the Mesozoic Fissure Fillings of South Wales.* Ph.D. thesis, Univ. Bristol (2004).

24. Luo, Z. X., Kielan-Jaworowska, Z., & Cifelli, R. Evolution of dental replacement in mammals. *Bulletin of the Carnegie Museum of Natural History.* **36**, 159-176 (2004).

25. Carey, J. R. & Judge, D. S. Longevity records: life spans of mammals, birds, amphibians, reptiles, and fish. *Monographs on Population Aging.* **8** (2000).

26. Tidière, M., Gaillard, J. M., Berger, V., Müller, D. W., Lackey, L. B., Gimenez, O., ... & Lemaître, J. F. (2016). Comparative analyses of longevity and senescence reveal variable survival benefits of living in zoos across mammals. *Scientific Reports*, **6**, 36361 (2016).
