# Supplementary material for: Reptile-like physiology in Early Jurassic stem-mammals
Source: Nat Commun. 2020 Oct 12;11:5121. doi: 10.1038/s41467-020-18898-4 (PMC7550344; doi:10.1038/s41467-020-18898-4)
Supplement: Supplementary file 3 — Reporting Summary [file 41467_2020_18898_MOESM3_ESM.pdf]

natureresearch

Corresponding author(s): Elis Newham, Pamela G Gill, Ian J Corfe

Last updated by author(s): August 14th, 2020

Reporting Summary

Nature Research wishes to improve the reproducibility of the work that we publish. This form provides structure for consistency and transparency in reporting. For further information on Nature Research policies, see Authors & Referees and the Editorial Policy Checklist .

Statistics

For all statistical analyses, confirm that the following items are present in the figure legend, table legend, main text, or Methods section.

n/a	Confirmed	
☐	☒	The exact sample size (*n*) for each experimental group/condition, given as a discrete number and unit of measurement
☒	☐	A statement on whether measurements were taken from distinct samples or whether the same sample was measured repeatedly
☐	☒	The statistical test(s) used AND whether they are one- or two-sided<br>*Only common tests should be described solely by name; describe more complex techniques in the Methods section.*
☐	☒	A description of all covariates tested
☐	☒	A description of any assumptions or corrections, such as tests of normality and adjustment for multiple comparisons
☐	☒	A full description of the statistical parameters including central tendency (e.g. means) or other basic estimates (e.g. regression coefficient) AND variation (e.g. standard deviation) or associated estimates of uncertainty (e.g. confidence intervals)
☐	☒	For null hypothesis testing, the test statistic (e.g. *F*, *t*, *r*) with confidence intervals, effect sizes, degrees of freedom and *P* value noted<br>*Give P values as exact values whenever suitable.*
☒	☐	For Bayesian analysis, information on the choice of priors and Markov chain Monte Carlo settings
☒	☐	For hierarchical and complex designs, identification of the appropriate level for tests and full reporting of outcomes
☐	☒	Estimates of effect sizes (e.g. Cohen's *d*, Pearson's *r*), indicating how they were calculated

Our web collection on statistics for biologists contains articles on many of the points above.

Software and code

Policy information about availability of computer code

Data collection	Synchrotron tomographic data: in-house software developed by beamline scientists at respective beamlines, for references see Methods. Micro-computed tomographic data: reconstructed using NRecon version 1.7.1.0/ datos|x rec.
Data analysis	Tomographic data was analysed using ImageJ/Fiji v1.52n, and Avizo 8.0/9.3 (Thermo Fisher Scientific). Phylogenetically informed analyses were performed using the "ape", "geiger", "nlme" and "phytools" packages in Rstudio v1.2.5001. All other statistical analyses were performed using Past 4.x v.4.0 and Microsoft excel.

For manuscripts utilizing custom algorithms or software that are central to the research but not yet described in published literature, software must be made available to editors/reviewers. We strongly encourage code deposition in a community repository (e.g. GitHub). See the Nature Research guidelines for submitting code & software for further information.

Data

Policy information about availability of data

All manuscripts must include a data availability statement. This statement should provide the following information, where applicable:

- Accession codes, unique identifiers, or web links for publicly available datasets
- A list of figures that have associated raw data
- A description of any restrictions on data availability

The tomographic data that support the findings of this study are available from the corresponding authors upon reasonable request. Virtual thin sections from which cementum increment counts were made are deposited in the University of Southampton's PURE data repository as DOI number D1506 (https://doi.org/10.5258/SOTON/D1506). The source data underlying figures 5,6 and 7 and S3, S4 and S5 are provided as a Source Data File. Physiological source data for charts and graphs are additionally provided in Supplementary Data files and Supplemental tables and were originally downloaded from the online database of the Max Planck Institute (https://www.demogr.mpg.de/longevityrecords/0203.htm), an Ecological Archives database (http://www.esapubs.org/archive/ecol/E084/094/metadata.htm), the AnAge database (https://genomics.senescence.info/species/), and taken from the literature (references in main text and Supplementary files).

Field-specific reporting

Please select the one below that is the best fit for your research. If you are not sure, read the appropriate sections before making your selection.

- [ ] Life sciences
- [ ] Behavioural & social sciences
- [x] Ecological, evolutionary & environmental sciences

For a reference copy of the document with all sections, see nature.com/documents/nr-reporting-summary-flat.pdf

Ecological, evolutionary & environmental sciences study design

All studies must disclose on these points even when the disclosure is negative.

Study description	Comparison of lifespan counted from tooth cementum growth increments, and bloodflow index Qi measured from femoral foramina, between mammaliaforms Morganucodon and Kuhneotherium and extant mammals and reptiles.
Research sample	206 dental mammaliaform fossils chosen based on visual assessment of cementum preservation. 6 Morganucodon fossil femora chosen based on completeness, 56 extant small mammal femora chosen to provide a statistically significant comparative sample.
Sampling strategy	Mammaliaform teeth and dentaries with relatively intact roots were prioritised, as mechanical damage can remove outer cementum layers and growth increments. The maximum possible sample from available teeth was chosen in order to maximise the amount of teeth found to have readable cementum increments upon scanning The most complete three femora available were used for statistically robust comparison with extant taxa.
Data collection	Data collected was tomographic images from synchrotron and lab X-ray sources. EN, PGG, PB, VF, DH, TK, AK, FM, AP, BP, PS, HS, PT & IJC performed the synchrotron experiments. EN, AK & IJC performed the microCT experiments.
Timing and spatial scale	The study compares ~200 million year old mammaliaforms with extant mammals and reptiles to compare estimates of metabolic proxies in fossils to known values in extant taxa. No spatial data was used.
Data exclusions	Flying, gliding and marine taxa were excluded from life history/body mass comparisons with mammaliaforms due to known differences between these and terrestrial taxa in relationship of lifespan:body mass (see Methods for detail) these exclusion criteria were pre-determined.
Reproducibility	Cementum increment counts were repeated by three independent observers and coefficient of variation precision calculations made from results (see Methods). Comparison between counts suggests high precision when compared to previous cementochronological studies.
Randomization	Samples were grouped by species, no randomisation was possible
Blinding	Blinding was used for cementum increment counting of multiple virtual thin sections per specimen by three independent observers. Blinding was not performed for measuring nutrient foramina as it involves objective measurements rather than the subjective increment counting of cementochronology.

Did the study involve field work? [ ] Yes [x] No

Reporting for specific materials, systems and methods

We require information from authors about some types of materials, experimental systems and methods used in many studies. Here, indicate whether each material, system or method listed is relevant to your study. If you are not sure if a list item applies to your research, read the appropriate section before selecting a response.

Materials & experimental systems

n/a	Involved in the study
[x]	[ ] Antibodies
[x]	[ ] Eukaryotic cell lines
[ ]	[x] Palaeontology
[x]	[ ] Animals and other organisms
[x]	[ ] Human research participants
[x]	[ ] Clinical data

Methods

n/a	Involved in the study
[x]	[ ] ChIP-seq
[x]	[ ] Flow cytometry
[x]	[ ] MRI-based neuroimaging

Palaeontology

Specimen provenance	All specimens were from existing collections of NHMUK and UMZC
Specimen deposition	All specimens were loaned from and returned to NHMUK and UMZC collections
Dating methods	No dating methods were used

- [ ] Tick this box to confirm that the raw and calibrated dates are available in the paper or in Supplementary Information.
