# Supplementary material for: Reptile-like physiology in Early Jurassic stem-mammals
Source: Nat Commun. 2020 Oct 12;11:5121. doi: 10.1038/s41467-020-18898-4 (PMC7550344; doi:10.1038/s41467-020-18898-4)
Supplement: Supplementary file 4 — Description of Additional Supplementary Files [file 41467_2020_18898_MOESM4_ESM.pdf]

Description of Additional Supplementary Files

File Name: Supplementary Data 1
Description: Table of independent cementum increment counts for data analysed by three observers.

File Name: Supplementary Data 2
Description: Table of *Morganucodon* and *Kuhneotherium* specimens with cementum increment counts, with fissure of origin and element details.

File Name: Supplementary Data 3
Description: Table of physiological data for extant taxa plus literature source references.
